# Predict Treatment Response by Magnetic Resonance Diffusion Weighted Imaging: A Preliminary Study on 46 Meningiomas Treated with Proton-Therapy

**DOI:** 10.3390/diagnostics11091684

**Published:** 2021-09-15

**Authors:** Paola Feraco, Daniele Scartoni, Giulia Porretti, Riccardo Pertile, Davide Donner, Lorena Picori, Dante Amelio

**Affiliations:** 1 Neuroradiology Unit, S. Chiara Hospital, Largo Medaglie d’Oro 9, 38122 Trento, Italy; 2 Department of Experimental, Diagnostic and Specialty Medicine (DIMES), University of Bologna, Via San Giacomo 14, 40122 Bologna, Italy; 3 Proton Therapy Center, APSS, Via al Desert 14, 38122 Trento, Italy; daniele.scartoni@apss.tn.it (D.S.); dante.amelio@apss.tn.it (D.A.); 4 Department of Radiology, S. Chiara Hospital, Largo Medaglie d’Oro 9, 38122 Trento, Italy; giulia.porretti@apss.tn.it; 5 Clinical and Evaluative Epidemiology Department—Trento Health Service, Via de Gasperi 79, 38122 Trento, Italy; riccardo.pertile@apss.tn.it; 6 Nuclear Medicine Unit, Santa Chiara Hospital, Azienda Provinciale per i Servizi Sanitari della Provincia Autonoma di Trento, 38123 Trento, Italy; davide.donner@apss.tn.it (D.D.); lorena.picori@apss.tn.it (L.P.)

**Keywords:** diffusion-weighted imaging, apparent diffusion coefficient, magnetic resonance imaging, meningiomas, proton therapy, treatment response

## Abstract

Objective: a considerable subgroup of meningiomas (MN) exhibit indolent and insidious growth. Strategies to detect earlier treatment responses based on tumour biology rather than on size can be useful. We aimed to characterize therapy-induced changes in the apparent diffusion coefficient (ADC) of MN treated with proton-therapy (PT), determining whether the pre- and early post-treatment ADC values may predict tumour response. Methods: Forty-four subjects with MN treated with PT were retrospectively enrolled. All patients underwent conventional magnetic resonance imaging (MRI) including diffusion-weighted imaging (DWI) at baseline and each 3 months for a follow-up period up to 36 months after the beginning of PT. Mean relative ADC (rADCm) values of 46 MN were measured at each exam. The volume variation percentage (VV) for each MN was calculated. The Wilcoxon test was used to assess the differences in rADCm values between pre-treatment and post-treatment exams. Patients were grouped in terms of VV (threshold −20%). A *p* < 0.05 was considered statistically significant for all the tests. Results: A significant progressive increase of rADCm values was detected at each time point when compared to baseline rADCm (*p* < 0.05). Subjects that showed higher pre-treatment rADCm values had no significant volume changes or showed volume increase, while subjects that showed a VV < −20% had significantly lower pre-treatment rADCm values. Higher and earlier rADCm increases (3 months) are related to greater volume reduction. Conclusion: In MN treated with PT, pre-treatment rADCm values and longitudinal rADCm changes may predict treatment response.

## 1. Introduction

Meningiomas (MN) are extra-axial tumours arising from the inner layer of the dura. They are the most common adult intracranial tumour, and most of them are benign [1]. The treatment strategy for MN includes observation, surgery, and radiation therapy [2]. Small asymptomatic MN can often be simply followed up by serial imaging, while the current standard of care for symptomatic or growing MN consists of maximal resection. In the case of partial excision, difficult surgical accessibility, recurrence, and higher-grade MN (WHO II and III), other kinds of treatments can be considered. These include photon-based fractionated radiotherapy, intensity-modulated radiotherapy, volumetric modulated arc therapy, and stereotactic radiotherapy [2,3]. However, alternative radiation techniques have emerged for treating MN and other tumours of the central nervous system. In particular, proton therapy (PT) is useful to treat lesions with difficult surgical access, such as skull base MN [4], making it possible to deliver high doses of radiation to the tumour with the relative sparing of adjacent tissues. Indeed, protons deliver a lower entry dose, depositing the majority of their energy at the end of their path, yielding a typical narrow dose energy peak called the “Bragg peak” [5]. This steep fall-off allows for the delivery of high doses and spares of healthy tissue beyond the tumour, reducing acute and late side effects. Usually, clinical response criteria after radiation treatment are based on tumour size stability or reduction at the follow-up MRI control after the end of the treatment (RANO criteria) [6]. However, a considerable subgroup of MN exhibit indolent and insidious growth. In these cases, other strategies to detect earlier treatment responses and to refine treatment decisions based on tumour biology rather than only on size can be useful [7]. 

In the last decade, it has been widely described how the use of diffusion weighted imaging (DWI) and apparent diffusion coefficient (ADC) maps may help in the evaluation of early tumour treatment response, particularly in those cases without tumour size changes [8,9,10]. In particular DWI can non-invasively provide direct insight into the microscopic physical properties of tissues through observing the Brownian movement of water and by reflecting cellularity within the lesions by means of the ADC values. Furthermore, since PT causes intra- and extra-cellular changes, which also modify the water molecule’s ability to diffuse into the tissue itself, the assessment of ADC maps could also be applied to screen for MN ultrastructure changes after treatment [8]. 

We aimed to characterize therapy-induced changes in MN during a follow-up period of 27–36 months after PT irradiation, determining whether the pre- and early post-treatment ADC values may predict tumour response. We hypothesized that MN receiving PT may show different longitudinal patterns of therapy-induced damage measured by means of ADC and that these changes can be related to specific MN pre-treatment characteristics. Information revealed by these patterns may be useful to predict the treatment response.

## 2. Materials and Methods

The ethics committee of our hospital (Azienda provinciale per i servizi Sanitari di Trento—Code A574) approved this retrospective study, and a detailed written informed consent form was signed by all subjects. 

### 2.1. Subjects

Sixty-seven patients who underwent active beam PT (Proteus^®^PLUS, IBA, Louvain-la-Neuve, Belgium) at our institution from March 2015 and February 2019 were selected. All of the patients received PT for residual, progressive, or non-operable lesions. Dose selection was based on the assessment of a variety of inter-related factors, including patient features (age, performance status) and tumour features (grading, location). For benign MN, total doses of 50–54 Gy (relative biological effectiveness [RBE]) were applied. Grade II lesions were treated with 60 GyRBE, while grade III tumours received a boost up to 66 GyRBE in case of a gross residual tumour. All of the treatments were delivered at 1.8–2 GyRBE per fraction. The inclusion criteria included (1) definite diagnosis (by histopathology, MRI, and DOTA0-D-Phe1-Tyr3-octreotide-(68) Ga-DOTATOC PET) of intracranial meningioma; (2) cMRI and DWI performed within 1 month before radiation therapy; and (3) follow-up cMRI and DWI performed after 3 months, 6–9 months, 12–15 months, and 21–36 months from the end of the treatment. Patients who underwent re-irradiation and those with an unmeasurable residual meningioma (e.g., meningeal thickening) were excluded. 

### 2.2. MR Imaging Techniques

MRI studies were performed on two different 1.5 Tesla magnets (The Optima™ MR450w,1.5T, ge, Milwaukee; Ingenia, 1.5T, Philps, The Netherlands). All of the patients received routine clinical MRI scans including axial T1-weighted fast spin echo (FSE), axial and coronal T2-weighted fast relaxation fast spin echo-propeller sequence (FRFSE-Propeller), axial fluid-attenuated inversion recovery imaging (FLAIR), and axial T2*-weighted gradient echo (GRE) with section thickness (5 mm), intersection gap (1 mm), and FOV (240 × 240 mm) uniform in all sequences. After IV contrast-agent injection (gadobutrol, 0.1 mmol/kg), a 3D fast-spoiled gradient-echo (FSPGR) sequence with isotropic voxel was acquired. 

On each scanner, the DWI acquisition consisted of a diffusion-sensitized axial 2D spin-echo sequence with EPI readout, with two b values of 0 and 1000 s/mm^2^. The section thickness was 4 mm with an intersection gap of 1 mm. The diffusion gradients were encoded in the x, y, and z directions to generate three sets of diffusion-weighted images. ADC maps were automatically calculated by the integrated scanner software and were converted into standard units (10^−3^ mm^2^/s). All of the images were assessed by a European board-certified neuroradiologist (PF) with 15 years of experience. 

### 2.3. Imaging Analysis

MR images were analysed on an off-line dedicated workstation (Advantage Workstation 4.3_8 GE). Each PT treatment plan was evaluated in order to the ability to assess the treated lesions. To ensure precise ROI placement on the solid tumour component, the DWI images were co-registered with conventional MRI (T1-weighted pre- and post-gadolinium and T2-weighted). Hence, the ADC values were measured by manually drawing tumour contours on the ADC maps on the section showing, the lower signal intensity area recording the ADCmean values of this ROI.

Eventually, to minimize variances in the ADCmean values, the relative ADCmean (rADCm) was obtained from the ratios of the tumour ADCm to the ADCm of a normal-appearing reference region (left cerebellum), defined on T2-weighted and contrast-enhanced T1-weighted images (CE-T1w).

**Figure 1:**
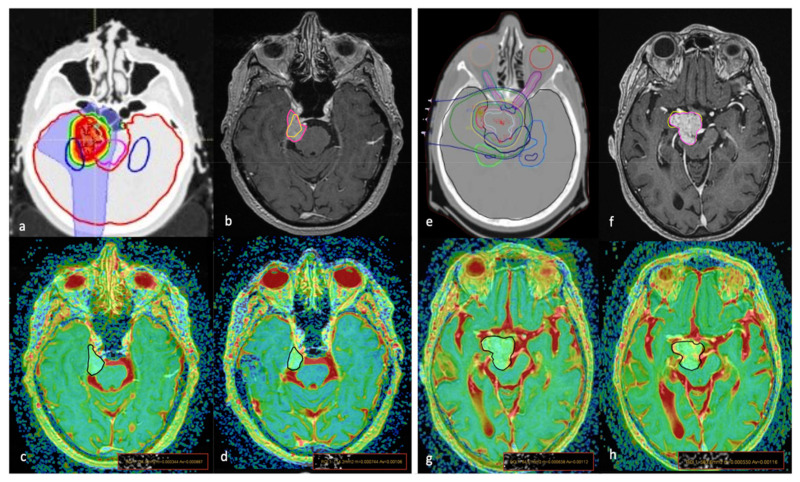
The proton therapy (PT) simulation planning computed tomography scan for target volume and organ at risk delineation (**a**,**e**); graphic representation of tumour volume variation (VV) on axial MRI T1-weighted + c images of two cases (**a**–**h**) treated with PT; areas inside the pink lines (**b**,**f**) represent the pre-treatment contour while areas inside the yellow lines represent the post-treatment (21 months) contour showing a reduction in (**b**) (VV = −46.9%) and mild increase in (**f**) (VV = +3.40%). The ROI placement on the co-registered T1w + c/ADC maps images, obtained before (**c**,**g**) and 24 months after (**d**,**h**) treatment, show lower pre-treatment values (**c**) in the first patient, with a significant increase after PT, while in the second patient had higher pre-treatment ADC values that did not change significantly during the follow-up exam.

Finally, for each MN, the volumes from the first and last available post-treatment scans were used to calculate the percentage variation of the tumour volume (VV-last volume-volume—pre-treatment volume/pre-treatment volume). The volumes were manually drowned on contrast-enhanced T1-weighted images by using a computerized image analysis tool (Raystation treatment planning system (TPS, Version 8.0) (Figure 1).

### 2.4. Statistical Analysis 

Statistical analyses were performed by using SAS System (9.1.3 version). Descriptive statistics included the mean and standard deviation of continuous variables and scores; in case of categorical parameters, observed frequencies and percentages distributions were used. The Wilcoxon–Mann–Whitney test was used to evaluate the differences in the mean rADCm values of the overall group between baseline and each post-treatment exam and among each post-treatment exam. These data were also controlled for the potential effects derived from the radiation dose by using a multiple regression analysis. Student’s *t*-test was used to evaluate the difference in the rADCm and VV among the groups divided according to the MN volumetric changes between baseline and follow-up (VV; threshold at −20%). Fisher’s exact test was then used to evaluate localization prevalence (skull base/convexity) in each group. Finally, Pearson’s correlation coefficient (r) was calculated to assess the relationships between the data (radiation dose, pre-treatment rADCm, rADCm variation and VV). A p < 0.05 was considered statistically significant for all the tests. 

## 3. Results

From the overall patient samples that were screened (*n* = 67), not all of them had a DWI sequence acquired at the 1-month pre-treatment exam (14) and/or MRI acquired in our institute (*n* = 7). As a result, 44 patients and 46 MN were included in the study. Patients were irradiated because of inoperable (25/46; 55%) or residual/recurrent (21/46; 45%) MN. The demographic characteristics and conventional MRI features of MN are shown in Table 1.

### 3.1. ADC Longitudinal Changes 

**Figure 2:**
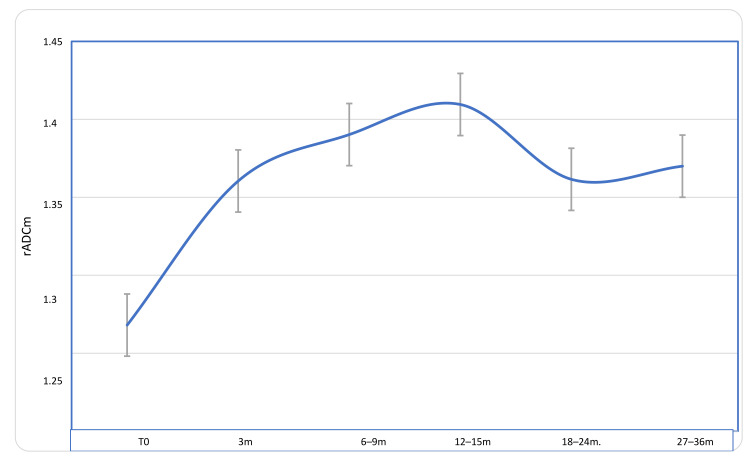
Longitudinal course of rADCm values in patients with MN during a follow-up of 27–36 months after proton-therapy treatment. A significant progressive increase of rADCm values was detected at each time point if compared to baseline, with a peak at 12–15 months; the values then slightly decrease but remain significantly higher than those at baseline.

At baseline, there was no difference in rADCm values between the subjects who had benn previously treated with surgery (20 out of 46) and the subjects who had not been previously treated (26 out of 46). In the overall group, the statistical difference between the pre-treatment rADCm and the last available post-treatment rADCm group was statistically significant (*p* = 0.0007), with higher rADCm values found in the last follow-up exam group. Compared with baseline, a significant progressive increase of rADCm values was detected at each time point (Table 2; Figure 2). 

### 3.2. Comparison of rADCm Values among Patients Grouped by Different Volume Changes 

Since the response criteria are based on the tumour volume variation [6], we decided to group patients on the basis of tumour VV. We set a VV threshold at −20%, evaluating longitudinal rADCm changes within groups.

Considering size from baseline (T0) to the last available follow-up exam, in 18 out of 46 MN (39.1%), the volume had a decrease greater that was than 20% of the baseline tumour volume (mean change = −26.6% ± 7.7%; range—46.8 to—20.01%), 4 out of 46 (8.7%) subjects showed an increased tumour volume from baseline to follow-up (mean increase = +42.7% ± 49.3; range +3.4% to + 114%), while 24 out 46 (52.1%) had a volume decrease of less than 20% of the baseline tumour volume (mean change = −11.37% ± 5.7%; range—1.3 to—19.23%). 

Since only four patients showed an increase in volume, we divided the subjects into two groups: Group-1: subjects showing a decrease greater than −20% of their baseline volume (*n* = 18), suggesting a good response to treatment; Group-2: subjects showing a decrease of less than −20% of their baseline volume or an increase (*n* = 28), suggesting a lack of response or only a moderate response to treatment. 

**Figure 3:**
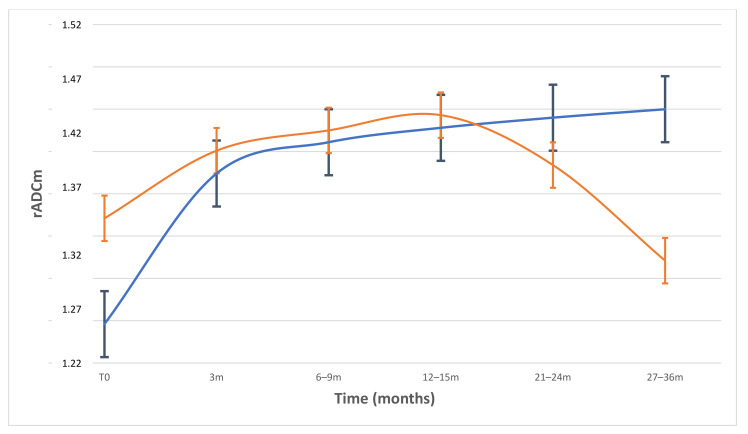
Plot of longitudinal course of rADCm values and standard mean error bars in patients divided by volume variation percentage (VV; threshold −20%). In patients with VV > −20% (blue line), rADCm levels increased continuously and showed significant differences from baseline to each FU (*p* < 0.001). In patients with lower VV < −20%) (orange line), rADCm values did not change significantly to baseline, showing a progressive reduction after 12–15 months FU.

A statistically significant increase in rADCm values from T0 to each follow-up MR was only found within Group-1 (*p* = 0.0001), whereas Group-2 did not exhibit similar findings. A significant difference in the baseline rADCm values was found between groups (p = 0.0018) with lower pre-treatment values in Group-1. The descriptive results of the longitudinal changes in the rADCm values in the two predetermined MN groups are summarized in Table 3 and Figure 3. There was no significant prevalence in tumour location among the groups. 

### 3.3. Correlations among rADCm Values, VV and Therapeutic Dose

**Figure 4:**
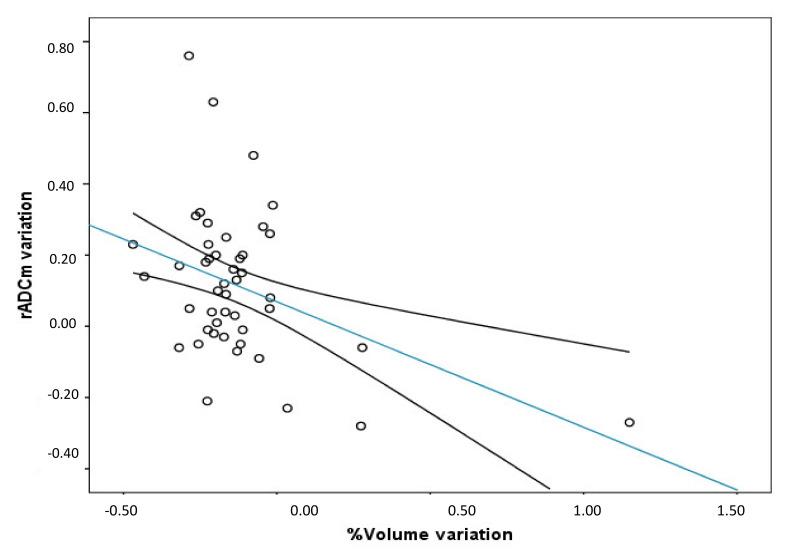
Between the variation of rADCm (Last rADC-Pre rADCm) and volume variation percentage. A significant negative correlation between the variables was found (r = −0.40; *p* = 0.0048; r^2^ = 0.21. (Trend line and mean confidence interval are shown).

A significant negative correlation between tumour VV and the variation of rADCm (last -rADC–pre rADCm) was found (r = −0.40; *p* = 0.0048) (Figure 4). No significant correlations were detected between the therapeutic dose (Gy), the pre-treatment rADCm, and the VV. 

## 4. Discussion

The clinical response criteria of MN after radiation treatment are usually based on tumour size stability or reduction on the follow-up control MRI after the end of the treatment (RANO criteria) [6]. However, a considerable subgroup of MN exhibit indolent and insidious growth [7]. In these cases, other non-invasive strategies able to predict treatment response or to detect earlier response, such as MR derived biomarkers, based on the evaluation of biological tumour characteristics rather than on size, can be useful. Our study revealed the utility of DWI in the detection of early tumour response and its ability to predict treatment outcome in MN patients treated with PT since MN structural changes induced by PT can be detected and quantified in vivo with DWI through ADC maps. It has been widely described how ADC maps are useful in the evaluation of early tumour treatment response, particularly in those cases without tumour size changes [8,9,10], but only a few works have reported on tumour response to PT due to the scarce availability of this treatment technique [11,12,13,14], only one if exclusively considering MN. In particular, Franconeri et al. describes early intra-treatment changes in MN treated with PT, but this study lacks follow-up data and long-term response parameters [11]. We retrospectively evaluated 46 MN during a follow-up period of up to 27–36 months. In the overall patient group, we found a significant and progressive increase of the rADCm in the post treatment exams compared to baseline, with a peak at the 12–15 months follow-up MR exam; the values then slightly decreased at 21–24 months (Figure 3). This result was expected; indeed, when tumours are treated with a range of anticancer therapies, such as PT, which induces cell death by apoptosis, necrosis, and cell lysis, there is an increase in the mobility of water in the tissue microenvironment, and the increase in water diffusion translates to an increase in the measured tissue ADC [15,16]. 

However, if we consider the changes in the rADCm values from baseline to the last follow-up control in the groups divided by VV, we can note that the pre-treatment rADCm values of Group-1 (the group with greater volume reduction, Table 3) were significantly lower when compared to those of Group-2 (*p* = 0.0018) and progressively increased during controls. These results are similar to other findings previously reported on rectal carcinoma, cerebral glioma, and hepatic metastases, which detected that tumours with lower baseline pre-treatment ADC values responded better to chemotherapy/radiotherapy treatment compared to tumours that exhibit high pre-treatment ADC values [17,18,19,20,21,22]. Although the exact nature of the types of damage and biological processes underlying PT are still subject of some debate, as a matter of fact, the effectiveness of radiation depends on DNA damage induction and processing [23] and on apoptosis induction in particular [24]. Different cells/tissues can show different levels of response that are linked to both the proliferation index and molecular pathways that regulate the apoptotic response after tissue, cellular, and genetic damage [25]. Hence, the intrinsic MN microarchitecture together with its molecular subtype can be responsible for the variability of radiation response. 

Several studies have described DWI capability of differentiate MN subtypes and their grading [26,27,28] to provide significant information about tissue microarchitecture (cell count and ki67-LI) by means of ADC [29,30].

In particular it has been shown that atypical/malignant MN have lower intra-tumoural ADC values than typical MN [27]. Indeed, high cell proliferation may lead to a higher cell density and, as a result, less stroma, both of which may cause more diffusion-restriction of water molecules, leading to lower ADC [31]. Thus, the different pre-treatment and longitudinal rADCm changes we found among groups supported the evidence that different subtypes of MN present different sensitivity to PT [25,32].

Moreover, if we evaluate the rADCm treatment-induced longitudinal changes in the two groups, different behaviours can be examined with specific regard to the 3-month and 12–15-month follow-up exams. 

Indeed, even if both groups had early rADCm value increases, Group-1 exhibited higher rADCm variation compared to baseline at 3 months than Group-2 (Table 3; *p* = 0.02), maintaining a progressive increase during follow-up exams. These specific early greater increases of rADCm values are potentially related to tumour that is more sensitive to PT treatment. In particular, we supposed that a more differentiated and cellular MN with a lower pre-treatment rADCm value is also more sensitive to PT treatment showing earlier treatment-induced changes than others. On the other hand, Group-2, which exhibited higher pre-treatment values, reached its peak values at 12–15 months, and those values then progressively decreased. It is known how the radio-sensitivity of a tumour is also based on its microenvironment, such vascularity or hypoxia, and high pre-treatment ADC values in tumours might reflect the presence of necrosis or the loss of cell membrane integrity [33]. Areas of necrosis within a tumour are often hypoxic, acidotic, and poorly perfused, leading to lower treatment sensitivity. Hence, higher ADC pre-treatment values among groups of MN patients may be predictive of a minor response to PT.

Finally, we found a negative correlation between the variation of rADCm (Last–pre rADCm) and tumour VV, as shown in Figure 4, in which we can observe that patients with higher rADCm variation (higher water diffusivity at the last follow-up exam) had higher volume reduction. This result is useful to support the role of ADC maps as a tool to also monitor the response in patients treated with PT.

We believe that our findings should lead to prospective studies regarding the standardised use of DWI and ADC maps in pre-treatment and follow-up exams to guide both the planning and management of patients with MN. In particular, our results may help the timing of follow-up MR exams with particular regards to those that occur 3 and 12 months after PT. Finally, MN showing high pre-treatment rADCm values and a not significant increase of rADCm values at 3 months after PT should be strictly monitored during the first year of follow-up since progressive stable or reduced rADCm values may be related to a subsequent volume increase.

## 5. Limitations

This study has some limitations that need to be acknowledged. The retrospective nature of the study did not permit control of the heterogeneity of MR imaging quality, which act as artefacts regarding ADC. The ADC measurement was performed as a single slide measurement and not as a whole lesion measurement, which could better reflect tumour biology. Further similar studies investigating histogram and texture analysis are needed to provide more and better results.

Finally, the basic description of the diffusion process assumed in DWI sequences does not permit the complete representation of the complexity underlying cellular components and structures that lead to a limited diffusion of water molecules. Actually, ADC values do not represent true tissue characteristics, since they are affected by both molecular diffusion and blood perfusion. Thus, through the use of more advanced MRI pulse sequences and a higher order of diffusion model (e.g., intravoxel incoherent motion analyses, which can separate the perfusion components from the true diffusion of water molecules), further studies may partially overcome these limitations [34].

## 6. Conclusions

Changes in DWI-derived parameters, such as ADC maps, in the target tumour maybe useful to monitor treatment response. The specific pre-treatment MN microstructure influences its secondary changes and the timing of the response to the PT. Given that the rADCm treatment-induced changes reflect radiation-induced histologic changes, this quantitative biomarker may be useful to monitor tumour structure and may have the potential to detect treatment responses earlier, refining treatment options based on tumour biology rather than those based exclusively on size [6].

## Figures and Tables

**Table 1:** Demographic characteristics and conventional MRI features of patient sample.

**Patients/MN**	44/46	
**Mean age ± SD**	65 ± 13.2	
**Sex (%)**	M	13 (29.5 %)
	F	33 (70.5 %)
**MN type (%)**	26 WHO I; 13 WHO II; 1 WHO III;	
**MN Location**	Convexity	13
	Skull base:	
	Cavernous sinus	13
	other (petroclival, sphenoid)	20

**Table 2:** The mean values of the rADCm at baseline (T0), 3, 6–9, 12–15, and 21–36 months in the overall group. A progressive and significant increase of rADCm values was detected at each time-point if compared to T0 rADCm (*p* < 0.05).

	T0	3 m	6–9 m	12–15 m	21–24 m	27–36 m
**rADC mean ± SD**	1.268 ± 0.245	1.360 ± 0.214	1.390 ± 0.224	1.409 ± 0.239	1.364 ± 0.251	1.378 ± 0.283

**Table 3:** Patients grouped by volume variation percentage (VV, threshold −20%). A significant difference in the baseline rADCm values was found between groups (*p* = 0.0018) with lower pre-treatment values detected in patients with higher VR (Group-1).

Groups	*n*	%	% of Volume Variation from Baseline to Last Follow-Up Exam (mean ± SD)	Pretreatment rADCm Values	% of rADCm Increase at 3 Months
Overall population	46	100	−12.54 ± 23.45	1.26 ± 0.24	8
Group-1	18	39,2	−26.3 ± 7.7	1.16 ± 0.20	16.3
Group-2	28	61.8	−3.65 ± 25.2	1.29 ± 0.23	6.1
*p*-value (Group 1–2)				**0.0018**	**0.02**

## Data Availability

The data that support the findings of this study are available from the corresponding author, [PF], upon reasonable request.
